# Low‐Load Blood Flow Restriction Training Enhances Brachial Blood Flow During Exercise but not Reactive Hyperemia in Experienced Climbers

**DOI:** 10.1111/sms.70211

**Published:** 2026-01-24

**Authors:** Titouan Paul Perrin, Hugo Randy, Pyrène Santal, Xavier Hugues, Nicolas Tourette, Marie Coudurier, Michel Guinot, Violaine Cahouet, Franck Quaine, Stéphane Doutreleau, Samuel Vergès, Laurent Vigouroux, Hugo Kerherve, Mathieu Marillier, Julien Vincent Brugniaux

**Affiliations:** ^1^ Inserm, CHU Grenoble Alpes, HP2 Univ. Grenoble Alpes Grenoble France; ^2^ CNRS, Grenoble INP, GIPSA‐Lab Univ. Grenoble Alpes Grenoble France; ^3^ CNRS, ISM Univ Aix‐Marseille Marseille France; ^4^ M2S laboratory Univ Rennes Rennes France

**Keywords:** blood flow restriction training, flow‐mediated dilation, near‐infrared spectroscopy, peak reactive hyperemia, regional blood flow, rock‐climbing

## Abstract

Low‐load blood flow restriction training (LLBFRT) induces significant vascular stress, which is often associated with vascular remodeling, increased capillarization and muscle blood flow. These adaptations may be of interest to climbers as their endurance performance is limited by blood supply due to the isometric and intermittent nature of finger flexors (FD) contraction. This study, thus, aimed to assess vascular adaptations to a 5‐week FD protocol using LLBFRT (*n* = 12, cuff pressure = 60% of the limb occlusion pressure) and compare these responses to those elicited by high‐load resistance training (HLRT, *n* = 12) and no specific training (CON, *n* = 12) in male climbers. Participants in LLBFRT and HLRT trained twice a week, respectively at ~40% and ~75% of maximal voluntary contraction (MVC). Before and after the intervention, flow‐mediated dilation (FMD), peak reactive hyperemia blood flow, brachial blood flow and muscle oxygenation (near‐infrared spectroscopy) at rest and during contractions from 10% to 60% MVC were assessed using brachial doppler‐ultrasound. Brachial blood flow across contraction intensities was significantly improved in LLBFRT (+19% ± 31%; *p* = 0.011, *d* = 0.5) but not in CON and HLRT. Oxy‐ and total hemoglobin concentrations decreased less during contraction following LLBFRT while did not change following CON and HLRT. Reactive hyperemia and FMD were not altered by any training modality. In conclusion, despite no difference with HLRT, LLBFRT was the only protocol which increased significantly blood flow of the feeding artery and O_2_ availability during finger flexor low‐intensity contractions. However, these adaptations were not accompanied by modifications of macrovascular structure or endothelial function.

## 1 Introduction

Rock‐climbing involves vertical movement that heavily engages upper limbs, particularly finger flexors (*flexor digitorum*: FD). These muscles must generate substantial force to grip holds and move between them. Falls may occur when FD can no longer produce the minimum force required, particularly due to fatigue during lead climbing. Finger flexors critical force (CF) (also named “endurance level” [1]), defined as the highest force that FD muscles can withstand without disrupting metabolic steady‐state [2], is therefore one of the main determinants of climbing performance [3, 4]. During FD rhythmic isometric contractions characterizing rock‐climbing [5], a primary limiting factor of CF is muscle blood flow, and therefore oxygen (O_2_) delivery [6, 7], owing to the intramuscular pressure that compresses the microvascular bed and to having the arm frequently positioned above the head [8]. This compression increases with contraction intensity, progressively reducing muscle blood flow [9, 10], and eventually leading to complete ischemia, a point called muscular occlusion threshold (MOT), which conceptually refers to the critical occluding tension calculated during dynamic contractions [11]. At intensities exceeding this threshold, O_2_ delivery is restricted to relaxation phases of these intermittent contraction cycles. Therefore, climbing requires specific vascular capacities to maintain an adequate blood supply during exercise, despite the contraction‐induced partial or complete ischemia.

Climbers are chronically exposed to important variation of shear stress and low O_2_ availability which are known to stimulate vasodilation and angiogenesis [12, 13, 14]. Compared to non‐climbers, they present with increased brachial artery diameter and muscle capillarization leading to greater limb blood flow during relaxation phases, post‐exercise recovery, and following ischemia [15, 16, 17]. Interestingly, such specific structural and functional adaptations are not associated with improved endothelial function compared to non‐climbers [17]. This phenomenon is called the athlete paradox [18] and may be due to decreased wall thickness and increased baseline dilation, hence limiting dilation capacity [18]. Specific training aiming at inducing FD vascular adaptations to improve muscle O_2_ delivery may therefore be of particular interest to increase climbers' CF. It remains unknown which climbing specific training is suitable to target such vascular remodeling.

Low‐load blood flow restriction training (LLBFRT) consists in exercising using a pneumatic cuff inflated at the proximal part of the active limb to reduce arterial inflow and block venous outflow [19]. Typically performed at low intensities, that is, ~20%–40% of the maximal voluntary contraction (MVC), this training modality reduces arterial inflow and greatly decreases—or even blocks–venous outflow. This alters O_2_ supply [20], impairs metabolite clearance [21, 22] compared to unrestricted training, and leads to a large hyperemic response during cuff deflation. These characteristics have been shown to improve vascular function and structure, specifically increasing: (i) artery diameter and limb blood flow during and after exercise or following arterial occlusion [23, 24, 25]; (ii) capillary filtration [24, 26] and capillarization [27, 28, 29]; (iii) endothelial function measured by flow‐mediated dilation (FMD) [30, 31, 32] even if the latter is not a universal finding [33, 34]. Vascular adaptations to LLBFRT have also been detected using near‐infrared spectroscopy (NIRS), through increased blood volume (Hbtot) during exercise associated with maintained O_2_ availability (HbO_2_) within the exercising muscle [35]. Training under blood flow restriction may, therefore, improve vascular function and structure and, in turn, enhance blood supply during exercise. These positive adaptations may be highly beneficial for climbers but chronic vascular responses to LLBFRT have never been specifically examined in this population. Notably, climbing and LLBFRT appear to induce similar vascular adaptations, and it remains unclear whether the combined stimulus of blood‐flow restriction and intermittent isometric climbing would result in additive vascular adaptations. As experienced climbers may already be highly adapted to the vascular stress imposed by climbing, it may be hypothesized that this combined stimulus would result in positive vascular adaptations.

The aim of this study was to evaluate the vascular adaptations (blood flow during exercise, reactive hyperemia, endothelial function) induced by 5 weeks of isometric FD training in LLBFRT and compare these responses to those elicited by high‐load resistance training (HLRT, commonly used to improve climbing performance) or no specific training (CON). We hypothesized that LLBFRT would improve blood flow at low contraction intensity and peak reactive hyperemia, reflecting structural vascular adaptations such as increased arterial diameter and enhanced capillarization, but would not significantly enhance FMD. In contrast, we postulated that HLRT and CON would induce minimal vascular changes, as climbers are already accustomed to these stimulus.

## 2 Methods

### 2.1 Participants

Thirty‐nine experienced male sport climbers were included in this study, of whom 36 completed the study, with three participants excluded due to injury during the training period. They were healthy young adults (aged 18–50 years), climbing at least twice per week with recent (last 3 months) self‐reported red‐point climbing ability greater than 18 on the international rock‐climbing research association (IRCRA) grading scale [36]. Participants were randomly divided into three groups: LLBFRT (*n* = 12), HLRT (*n* = 12) and a control group (CON, *n* = 12). Baseline demographic and climbing characteristics of the participants are shown in Table 1. A flow chart of the study is presented in Figure S1. Participants gave their written informed consent to participate in this study which was approved by the French Ethics Committee for Research in Sports Science (IRB00012476‐2023‐10‐05‐246) and complied with the Declaration of Helsinki (2008).

**TABLE 1:** Participants' demographic and climbing characteristics (completers only).

	CON	HLRT	LLBFRT	*p*
Height (m)	1.77 ± 0.07	1.77 ± 0.06	1.80 ± 0.07	0.611
Body mass (kg)	68.3 ± 6.7	68.3 ± 6.3	71.5 ± 7.7	0.417
Age (years)	30 ± 7	28 ± 5	25 ± 4	0.172
Training sessions per week	3 ± 1	4 ± 2	3 ± 1	0.318
IRCRA max score	21 [20; 22]	21 [21; 24]	21 [20; 23]	0.729
IRCRA lead score	21 [20; 22]	21 [21; 24]	20 [19; 22]	0.480
Finger flexors MVC (N)	361 ± 67	370 ± 65	378 ± 64	0.690
FD adipose tissue thickness (mm)	2.8 ± 0.3	2.9 ± 0.6	2.9 ± 0.4	0.868

*Note:* Data are presented as mean ± SD or median [1st quartile; 3rd quartile]. *p*‐values refer to the group effect following ANOVA or Mann–Whitney *U* tests.

Abbreviations: CON, control group; FD, fingers flexors; HLRT, high‐load resistance training group; IRCRA, international rock‐climbing research association; LLBFRT, low‐load blood flow restriction training group; MVC, maximal voluntary contraction.

### 2.2 Experimental Design

The week before (PRE) and after the training intervention (POST), participants came to the laboratory for the FMD test, the evaluation of the FD MVC, and eight 30‐s FD contractions from 10% to 70% MVC on a customized force ergometer. PRE and POST measurements were conducted at a similar time of day, with participants instructed to refrain from any intense exercise 24 h prior and from the consumption of alcohol, caffeine and tobacco on the day of experiment.

#### 2.2.1 Training Intervention

Participants in LLBFRT or HLRT underwent supervised isometric intermittent FD resistance training twice per week for 5 weeks. Participants were instructed to continue with their usual climbing routine without increasing or decreasing their typical training regimen and adding fingerboard exercises. Both exercises consisted in three sets of repetitive 10‐s intermittent contractions using both hands (with feet on the ground and weighted if the target force was greater than body weight), interspaced by 6‐s rest. The target force was relative to the FD MVC assessed in PRE, and reassessed at the beginning of the third and eighth training session. Participants in CON were asked to keep on with their climbing routine during the 5 weeks of intervention without adding any FD specific training. Training sessions were conducted on a 12 mm‐deep hold of a specific hangboard equipped with strain gauges sampling at 50 Hz (SmartBoard, Peypin d'Aigues, France). The vertical force applied to the holds was directly displayed on a smartphone allowing participants to adjust the force output in order to reach the target.

#### 2.2.2 HLRT Modality

The present study uses the same protocol as published elsewhere, which was designed to increase both FD strength and endurance [1]. A typical training session consisted of three sets of intermittent contractions performed at ~65%–80% MVC on the instrumented hangboard and interspersed by 8 min of passive recovery. Each set consisted of 10 to 12 contractions and was designed to bring participants to exhaustion, that is, the point where they were no longer able to maintain at least 90% of the target force. The target force was individually adjusted in each session by ±2.5% MVC steps when the participant was able to sustain the target force during 12 repetitions or < 8 repetitions for each set.

#### 2.2.3 LLBFRT Modality

The protocol included three consecutive sets, separated by 1 min of passive recovery. The first set consisted of 20 contractions and the following two of 14 contractions. Similar to the HLRT group, the session was designed to bring participants to exhaustion. Target force (~30%–50% MVC) was individually adjusted between sessions by 2.5% MVC steps when the participant was able to maintain the force until the end of the last set or when the climber was unable to reach the target for five or more repetitions before the end of the set. Blood flow was restricted in each arm using a 10‐cm pneumatic cuff—with an 8‐cm wide bladder—wrapped around the proximal part of the arm. Individual limb occlusion pressures (LOP) were automatically determined by the device (MAD‐UP Pro, MAD‐UP SAS, Angers, France) at the beginning of each training session on a relaxed lying supine position. The cuff pressure was set to 60% LOP and inflated 30 s before the start of the session and deflated 15 s after the end of the last contraction. Cuff pressure during exercise was automatically adjusted by the device in order to avoid pressure spike due to muscle contraction [37].

### 2.3 Experimental Visits

During the whole visit, participants were lying supine on an auscultation bed, with their right arm extended and placed horizontally on a test table, at an angle of 90° from the torso, palm upwards. A doppler ultrasound system (Terason 3200 t, Teratech Corporation, Burlington, VE, USA) with a 15–4 MHz linear array transducer secured with a stereotactic adjustable clamp (MP‐177 PH0001, Hitachi‐Aloka Medical Ltd., Tokyo, Japan) was used in duplex mode to assess: (i) brachial FMD and peak reactive hyperemia blood flow following 5 min of forearm arterial occlusion; and (ii) blood flow during contractions from 10% to 70% MVC. All measurements on brachial artery were conducted by the same experienced operator. A second experienced operator conducted ulnar artery blood flow measurements during the contractions (details provided below).

#### 2.3.1 Flow‐Mediated Dilation

After 10 min of lying supine rest in a quiet room, FMD of the right brachial artery was measured. After a 1‐min baseline measurement, a pneumatic cuff (SC5D cuff connected to E20 Rapid cuff inflator and AG101 Cuff Inflator Air Source, Hokanson, Bellevue, WA, USA) positioned around the proximal part of the forearm, was inflated to a suprasystolic pressure of 250 mmHg for 5 min and then quickly released. Brachial artery diameter and blood flow velocity were continuously recorded from baseline until 3 min post‐deflation.

#### 2.3.2 Force Assessment

Five minutes following the FMD test, participants conducted an individualized FD warm‐up, including specific FD contractions of increasing intensity from 30% to 90% of their estimated maximal force. Climbers were given three attempts to apply their maximal force on the hold with the right hand during ~4 s, with a 1‐min recovery between two contractions. The FD MVC was calculated as the 0.5‐s maximal value of the best trial. After 5 min of recovery, a “warmed‐up” baseline brachial blood flow measurement was conducted. Then, participants conducted 30‐s contractions at constant intensity from 10% to 70% MVC in 10% increments, during which blood flow was continuously measured on brachial artery—using a fixed sample volume of 2 mm—and FD oxygenation monitored by NIRS (PortaLite, Artinis, Medical System, Elst, The Netherlands). Each contraction was interspersed by at least 3 min of passive recovery.

To determine the MOT with a 5% accuracy, a second operator measured ulnar blood flow during contraction, on the distal part of the forearm close to the head of the *Ulna*. Ulnar artery course into the forearm passes through *flexor digitorum profundus* and *superficialis* and should be compressed by intramuscular pressure during FD contraction. The MOT was therefore considered as the first contraction intensity from which there was no more ulnar blood flow during the last 15 s of the contraction. Due to the technical difficulties in obtaining a clear and continuous measurement of blood flow on this small artery, the operator was only asked to determine whether blood was flowing or not. After MOT was determined, participants were asked to conduct an extra contraction at an intensity corresponding to 5% MVC below the contraction that generated the occlusion, to improve MOT accuracy to within 5%.

MVC assessment and each contraction during experimental visits were conducted in a half‐crimp position, on a small 12 mm‐deep hold positioned just above the participant's hand placed on a supine position. The hold was equipped with a tri‐axial Kistler force sensor (9327 CU, Kistler Group, Winterthur, Switzerland). Force signal was amplified (Kistler 5070A) before being transmitted to a PowerLab system (16/30—ML880/P, ADInstruments, Bella Vista, Australia) and displayed at a sampling frequency of 1 kHz on the LabChart 7 software (ADInstruments). Participants had direct visual feedback on their force curve. To conduct the 30‐s continuous contractions, they were asked to maintain the force on a zone visually delimited on the computer screen as the force target ±2.5%. Half of our participants could not maintain 70% MVC during 30 s. This contraction intensity was therefore removed from analysis.

To determine muscular blood flow (mBF) by NIRS at rest and during recoveries, ~5‐s venous occlusions (75 mmHg) using the same cuff system than for FMD measurements, were performed 5 min after the FD MVC assessment (three repetitions), and 10 s after the end of each contraction.

#### 2.3.3 NIRS Measurement

During experimental sessions, changes in oxyhemoglobin (HbO_2_), deoxyhemoglobin (HHb), total hemoglobin (Hbtot) and the tissue saturation index (TSI) of the right FD were continuously monitored using a portable, continuous wave multi‐distance NIRS device. This system includes a single detector that received near‐infrared light at approximately 752 and 840 nm emitted by three sources placed at 30, 35 and 40 mm from the detector. The differential path length factor was fixed at 4. The probe was positioned on the muscle belly of the forearm FD, on a virtual line drown from the medial epicondyle of the humerus to the base of the carpus (lunate) proximal to the ring finger, 33% distal to the epicondyle. Double‐sided tape and an opaque plastic covering were used to ensure secure placement and prevent interference from ambient light. Due to the measurement depth of the NIRS device (1.5–2 cm), it was not possible to distinguish between signals from the FD *superficialis and profundus*; both are referred as “FD” in this study. Adipose tissue thickness (ATT) was measured by ultrasound before positioning the NIRS device during PRE measurements. As values were low (~3 mm) and did not differ between groups (Table 1), ATT was considered to marginally affect our result [38] and was therefore neglected.

Data were recorded continuously throughout the test at 25 Hz. Baseline tissue oxygenation was measured during 1 min on a lying supine position, 5 min after FD MVC assessment. Concentrations of each chromophore at each depth (one per optode) were averaged for analysis. During each contraction, variations from the 5th to the 25th s of contraction were calculated from each NIRS‐derived parameter (HHb, HbO_2_, tHb, and TSI). Analyses were not conducted during recovery periods due to movement artifacts.

Our estimate of mBF was based on the ∆Hbtot during the venous occlusion. Briefly, a cuff pressure of ~75 mmHg occludes venous outflow but only reduces arterial flow, resulting in an increased venous volume at a rate proportional to arterial inflow [39, 40, 41]. It has been demonstrated that calculating ∆Hbtot on a duration longer than the first cardiac beat following cuff inflation underestimate mBF [39]. As we were unable to determine cardiac beats on Hbtot curves precisely following intense contractions for some participants, we calculated ∆Hbtot during the first second of the venous occlusion. Concentration changes of [Hbtot] expressed in μmol.s^−1^ were then converted to ml blood per minute using the following formula [40]: mBF (mL.min^−1^) = (60 × ∆[Hbtot])/*C*, where *C* represents the average total hemoglobin sub‐units concentration, for which we assumed a value of 8.5 mmol L^−1^ [40, 41].

Two participants (one from HLRT and LLBFRT groups) were removed from NIRS analysis due to technical connection issues during the experimental visit.

#### 2.3.4 FMD% and Blood Flow Calculations

Throughout the entire FMD tests and during blood flow measurements, videos of the echograph display were recorded at 30 Hz using a dedicated software (Camtasia, TechSmith Corporation, East Lansing, MI, USA) and saved for future analyses that were conducted offline using an edge detection and wall tracking software (Bloodflow Analysis; National instruments, Austin, TX, USA). The operator was blinded from group allocation to remove investigator bias. B‐mode images allowed to continuously determine artery diameter. The blood‐velocity (cm.s^−1^) over a cardiac cycle was directly computed by the software from the doppler flow velocity spectrum. The flow mediated dilation score (FMD%) was calculated as the maximal diameter change (expressed in percentage) from baseline to peak dilation during the post deflation reactive hyperemia. To account for inter‐individual baseline diameter, which influences strongly FMD%, FMD% was allometrically scaled [42]. Shear rate was automatically calculated as [(4 × velocity)/diameter]. The total shear rate that contributed to the peak vasodilation was calculated as the area under the shear rate curve (SR_AUC_) from cuff deflation to peak dilation. Blood flow (mL/min) over a cardiac cycle was directly calculated by the software as (blood velocity × πr^2^) × 60, where *r* is the radius of the brachial artery lumen. Reactive hyperemia was determined as the peak blood flow averaged over a cardiac cycle post cuff deflation during the FMD test [43, 44]. During the contraction part of the experiment, baseline blood flow was averaged over the 10 highest consecutive cardiac cycles in a 1‐min window. The five highest consecutive cardiac cycles of the last 15 s of exercise were used to compute blood flow during contraction. The PRE to POST variation in brachial blood flow (∆blood flow), blood velocity (∆blood velocity) and artery diameter (∆artery diameter) were calculated as: ∆ = POST—PRE values. One participant from HLRT group was removed from FDM and RH analysis due to absence of blood flow occlusion during ischemia (visually detected by an increase in HbO_2_ during the occlusion).

### 2.4 Statistical Analysis

This research was part of a larger project aiming at investigating the muscular adaptations to FD LLBFRT in climbers. LLBFRT typically induces a close or slightly lower MVC improvement than HLRT, which improved FD MVC by 12.4% ± 8.4% after 4 weeks in climbers [1]. In this context, our sample size calculation was based on an expected 10% ± 10% gain in MVC measured on the SmartBoard in POST compared to PRE in LLBFRT. Using an intraindividual design, a two‐tailed test of significance, and *α* = 0.05, 11 participants per group were required with a statistical power of 0.9.

Statistical analyses were performed using RStudio (R‐Studio Inc., Boston, MA). Normality of the data distribution was assessed using Shapiro–Wilk tests and QQ plots and were Log‐ or Square Root‐transformed if necessary. Initial participants' characteristics were compared between groups using simple analyses of variance (ANOVA) or Mann–Whitney U test for continuous and discrete measurements respectively. To compare vascular adaptations depending on the training group, two types of analysis were conducted: (i) for data extracted from the FMD test (FMD%, peak reactive hyperemia) and for the MOT, two‐way, repeated measures ANOVA were conducted ([time (PRE vs. POST) × training modality (HLRT vs. LLBFRT vs. CON)]); (ii) for data extracted from contractions phases (brachial artery diameter, blood velocity and blood flow, NIRS variations during contractions), a contraction intensity effect had to be included in the statistical model. Two‐way, repeated measures ANOVA were conducted ([time (PRE vs. POST) × contraction intensity (0 vs. 10 vs. 20 vs. 30 vs. 40 vs. 50 vs. 60% MVC)]) for each training modality. For NIRS data, contractions intensities did not include the 0% MVC as we were interested in variations during contractions. Comparisons across training modalities were conducted using a single way ANOVA training modality (CON vs. HLRT vs. LLBFRT) based on PRE to POST variations (e.g., ∆blood flow) with all contraction intensities averaged as no time × contraction intensity interaction was reported. Allometrically scaled FMD% were analyzed via a mixed two‐way ANOVA with the diameter change on the natural log scale (ln(peak diameter) – ln(baseline diameter)) as the dependent variable, group and visit as fixed factors, and the log‐transformed baseline diameter (ln (baseline diameter)) as covariate. ANOVA were followed, when applicable, by pairwise *t*‐tests post hoc with holm correction. Effect sizes were evaluated using partial eta‐squared ($\eta_{p}^{2}$) for ANOVA and Cohen's *d* for parametric paired comparisons. They were interpreted according to Cohen's scale (small effect: 0.01 <$\;\eta_{p}^{2}$ < 0.06 or 0.2 < *d* < 0.5; moderate effect: 0.06 < $\eta_{p}^{2}$ < 0.14 or 0.5 < *d* < 0.8; large effect: $\eta_{p}^{2}$ > 0.14 or *d* > 0.8) [45]. Alpha level was set at 0.05.

## 3 Results

### 3.1 Flow‐Mediated Dilation (%) and Peak Reactive Hyperemia

There was no significant effect of training modality, of time or time × training group interaction for (i) baseline diameter; (ii) peak diameter; (iii) FMD% (Figure S1A); (iv) FMD corrected by SR or (v) reactive hyperemia (Figure S2B). However, time to peak dilation and SR_AUC_ were significantly greater in POST independently of the training group with small effect sizes (time to peak: *d* = 0.38; SRAUC: *d* = 0.48) (Table 2).

**TABLE 2:** Flow‐mediated dilation test across training modalities and time points.

	Time	CON	HLRT	LLBFRT	ANOVA effects ($\eta_{p}^{2}$)		
					Training modality	Time	Interaction
Baseline diameter (cm)	PRE	0.429 ± 0.047	0.441 ± 0.025	0.436 ± 0.050	0.607 (0.031)	0.334 (0.029)	0.161 (0.108)
	POST	0.43 ± 0.041	0.451 ± 0.021	0.433 ± 0.052			
Peak diameter (cm)	PRE	0.467 ± 0.048	0.478 ± 0.027	0.476 ± 0.054	0.678 (0.024)	0.149 (0.064)	0.14 (0.116)
	POST	0.468 ± 0.043	0.489 ± 0.027	0.474 ± 0.050			
Delta diameter (cm)	PRE	0.038 ± 0.022	0.037 ± 0.014	0.040 ± 0.016	0.862 (0.009)	0.537 (0.012)	0.983 (0.001)
	POST	0.039 ± 0.015	0.038 ± 0.015	0.041 ± 0.011			
FMD (%)	PRE	9.00 ± 4.90	8.33 ± 3.30	9.18 ± 3.47	0.747 (0.018)	0.622 (0.008)	0.912 (0.006)
	POST	9.06 ± 3.31	8.47 ± 3.06	9.71 ± 3.04			
Allometrically scaled FMD (%)	PRE	8.65 ± 0.95	8.49 ± 0.99	9.09 ± 0.95	0.879 (0.008)	0.444 (0.020)	0.955 (0.003)
	POST	8.80 ± 0.95	8.92 ± 1.00	9.54 ± 0.95			
Time to peak (s)	PRE	46.0 ± 11.0	46.4 ± 11.9	57.5 ± 16.9	0.093 (0.138)	**0.029** **(0.141)**	0.54 (0.038)
	POST	50.5 ± 9.8	55.4 ± 15.3	60.2 ± 16.4			
SR_AUC_ (A.U)	PRE	28 200 ± 9940	26 000 ± 6580	36 100 ± 14 200	0.052 (0.168)	**0.008** **(0.198)**	0.752 (0.018)
	POST	33 800 ± 7780	30 700 ± 8650	38 900 ± 10 800			
FMD corrected by SR (A.U)	PRE	0.322 ± 0.172	0.344 ± 0.186	0.285 ± 0.120	0.700 (0.022)	0.129 (0.071)	0.810 (0.013)
	POST	0.273 ± 0.095	0.301 ± 0.158	0.270 ± 0.116			
Peak reactive hyperemia (ml/min)	PRE	999 ± 284	930 ± 149	1000 ± 220	0.924 (0.005)	0.124 (0.075)	0.458 (0.049)
	POST	999 ± 319	1030 ± 176	1040 ± 306			

*Note:* Data are mean ± SD. ANOVA effects are presented as *p*‐value (partial‐eta squared); Pairwise *t*‐tests with holm correction were conducted to evaluate time effect for each training modalities, and differences of adaptations between groups. Values are bolded when significant.

Abbreviations: CON, control group; FMD, flow‐mediated dilation; HLRT, high‐load resistance exercising group; LLBFRT, low‐load blood flow restricted group; SR_AUC_, area under the curve of the shear rate.

### 3.2 Vascular and Muscular Responses to Exercise

Mean MOT and FD MVC did not change from PRE to POST, irrespective of the training modality (Table 3, Figure S3A,B).

**TABLE 3:** Strength and muscular occlusion threshold responses to training.

	Time	CON	HLRT	LLBFRT	ANOVA effects ($\eta_{p}^{2}$)		
					Training modality	Time	Interaction
MOT (%MVC)	PRE	39 ± 11	44 ± 9	39 ± 11	0.484 (0.04)	1 (0)	0.504 (0.04)
	POST	38 ± 12	42 ± 11	42 ± 12			
MOT (N)	PRE	137 ± 36	163 ± 40	146 ± 38	0.131 (0.12)	0.579 (0.01)	0.723 (0.02)
	POST	133 ± 33	169 ± 58	156 ± 46			
FD MVC (N)	PRE	361 ± 68	370 ± 65	378 ± 64	0.696 (0.02)	0.22 (0.05)	0.068 (0.15)
	POST	363 ± 63	396 ± 68	372 ± 45			

*Note:* Data are mean ± SD. ANOVA effects are presented as *p*‐value (partial‐eta squared).

Abbreviations: ANOVA, analysis of variance; CON, control group; FD MVC, finger flexors maximal voluntary contraction; HLRT, high‐load resistance exercising group; LLBFRT, low‐load blood flow restricted group; MOT, muscular occlusion threshold determined by ulnar doppler.

#### 3.2.1 Brachial Artery Measurements

**FIGURE 1:**
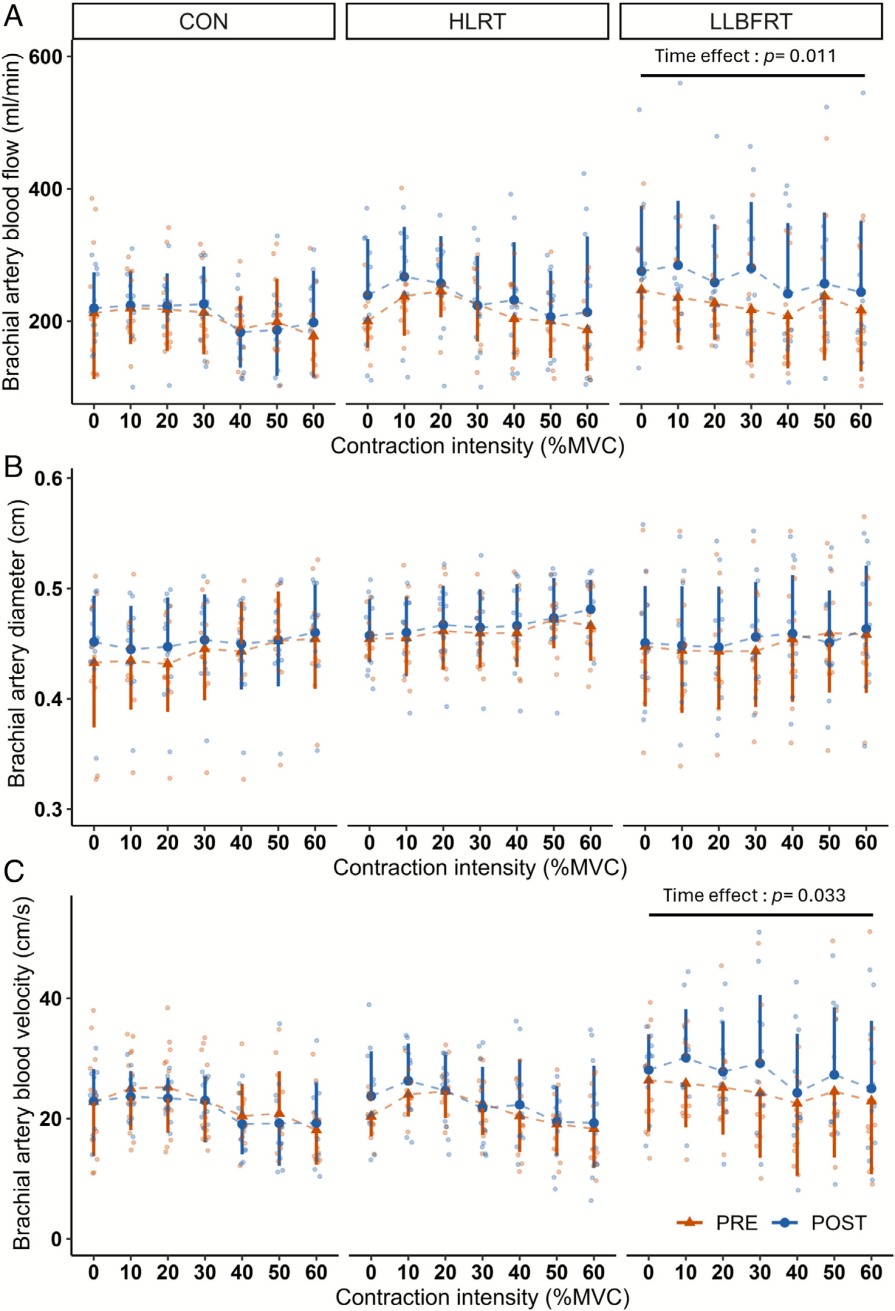
Effect of training on brachial artery blood flow (A) diameter (B) and blood velocity (C) during contractions from 10% to 60% MVC. Data are presented as mean ± SD. Orange triangles and blue circles respectively depict PRE and POST data; individual responses are presented using smaller color dots (orange: PRE; blue: POST). CON, control group; HLRT, high‐load resistance training; LLBFRT, low‐load blood flow restricted training; MVC, maximal voluntary contraction; PRE, before training protocol; POST, after training protocol.

Regardless of training modality, there was not any significant time × contraction intensity interaction on brachial artery blood flow, diameter and blood velocity. There were significant time effects for both brachial artery blood flow (*p* = 0.011) and blood velocity (*p* = 0.033) measured during contractions in LLBFRT, which respectively increased by +19% ± 31% (*d* = 0.50) and +16% ± 30% (*d* = 0.41) in POST compared to PRE (Figure 1). However, there was no significant effect of time on artery diameter (*p* = 0.184). In HLRT and CON, there were no time effects for either brachial artery blood flow, artery diameter, or blood velocity, which were therefore not significantly altered in POST compared to PRE (Table S1).

Considering differences between groups, there was a significant effect of training modality on PRE to POST ∆blood flow (*F* = 3.77, *p* = 0.024, $\eta_{p}^{2}$ = 0.03) and ∆blood‐velocity (*F* = 5.33, *p* = 0.005, $\eta_{p}^{2}$ = 0.041). Both were greater in LLBFRT compared CON (∆blood flow: +36 ± 71 vs.+5 ± 78 mL/min, *p* = 0.020; ∆blood‐velocity: +2.9 ± 7.0 vs. −0.7 ± 8.2 cm/s, *p* = 0.009) with no difference between LLBFRT and HLRT (∆blood flow: +36 ± 71 vs. +20 ± 71 mL/min, *p* = 0.331; ∆blood‐velocity: +2.9 ± 7.0 vs. +1.2 ± 6.0 cm/s, *p* = 0.304). The PRE to POST ∆artery diameter (*F* = 1.30, *p* = 0.274, $\eta_{p}^{2}$ = 0.01) was not different between training modalities.

#### 3.2.2 NIRS Assessment

**FIGURE 2:**
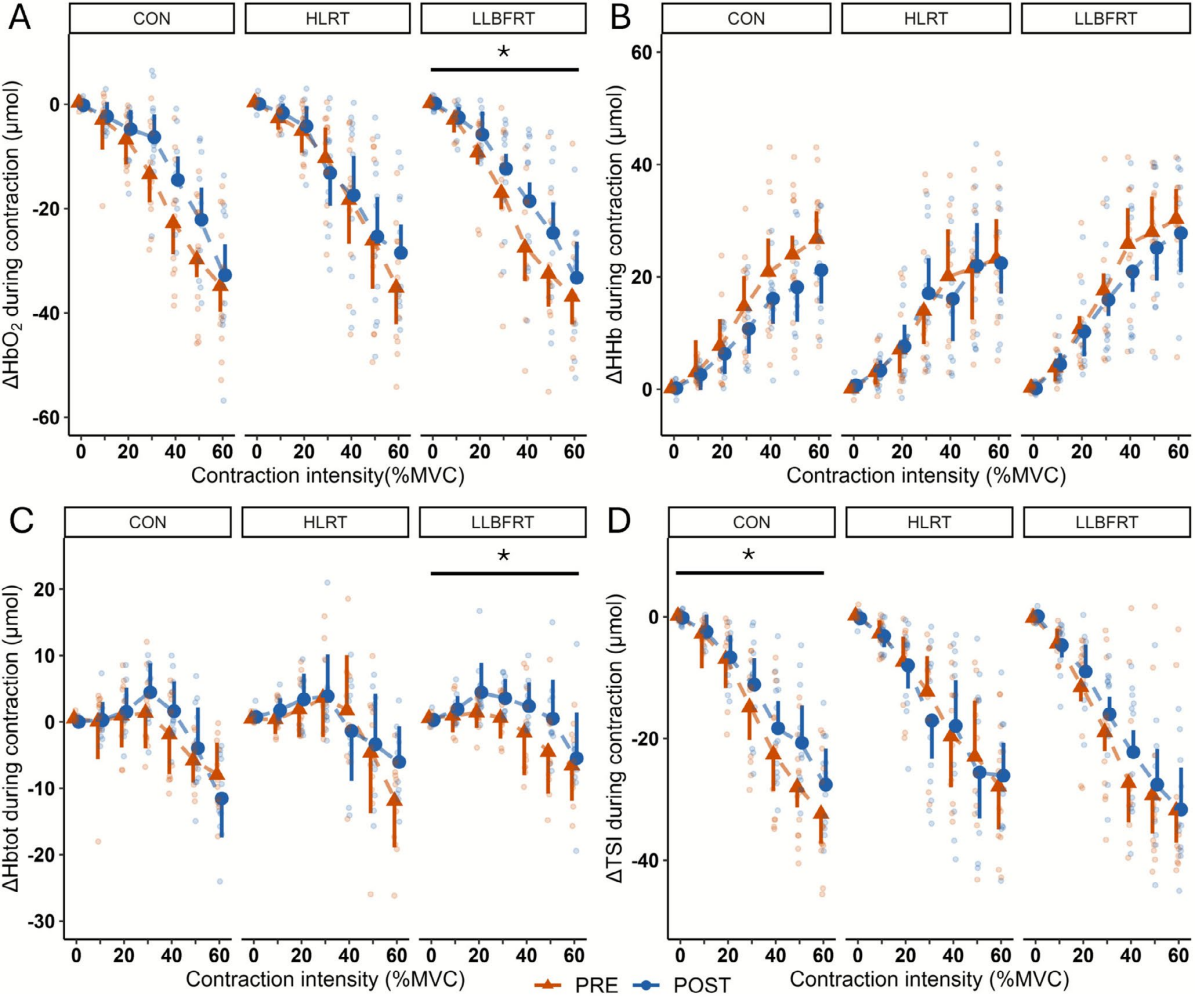
Effect of training and contraction intensity on ∆ HbO_2_ (A), ∆ HHb (B), ∆ Hbtot (C) and ∆ TSI (D) during contractions from 10% to 60% MVC. Data are presented as mean ± SD. Orange triangles and blue circles respectively depict PRE and POST data; individual responses are presented using smaller color dots (orange: PRE; blue: POST). CON, control group; HbO_2_, oxyhemoglobin; Hbtot, total hemoglobin; HHb, deoxyhemoglobin; HLRT, high‐load resistance training; LLBFRT, low‐load blood flow restricted training; POST, after training protocol; PRE, before training protocol; TSI, tissue saturation index; *p < 0.05 (time effect).

NIRS individual responses during contractions from 10% to 60% MVC are presented in Figure 2. Mean values with ANOVA results are presented in Table S2. Briefly, significant time × contraction intensity interactions were detected on HbO_2_ and Hbtot in HLRT (*p* = 0.001 and *p* = 0.007) and on Hbtot in CON (*p* = 0.009). However post hoc revealed no significant differences between PRE and POST regardless of contraction intensity (all *p* > 0.078).

In LLBFRT, there were significant time effects on HbO_2_ (*p* = 0.023) and Hbtot (*p* = 0.009). Regardless of contraction intensity, HbO_2_ (+4.3 ± 8.3 μmol, *d* = 0.52) and Hbtot (+2.8 ± 5.4 μmol, *d* = 0.52) were greater during contraction in POST compared to PRE.

In CON, there was a significant effect of time on TSI (*p* = 0.014). Regardless of contraction intensity, TSI (+3.3% ± 6.9%, *d* = 0.49) decreased less during contraction in POST compared to PRE.

#### 3.2.3 Muscular Blood Flow Post Contraction

Regardless of the training modality, mBF increased with contraction intensity (all *p* < 0.001) without any effect of time (CON: *p* = 0.543; HLRT: *p* = 0.228; LLBFRT: *p* = 0.518). A time × contraction intensity interaction was detected on mBF only in CON (*p* = 0.046; HLRT: *p* = 0.410; LLBFRT: *p =* 0.359) but without any significant post hoc (Table S2).

## 4 Discussion

This study is the first to examine the effects of 5 weeks of climbing‐specific LLBFRT on vascular structure and function in advanced climbers. We reported that (i) LLBFRT was the only intervention to enhance brachial blood flow and increase both oxyhemoglobin and total hemoglobin concentrations during contraction; (ii) these adaptations were greater than in CON (for brachial blood flow) but not significantly different from HLRT; and (iii) whatever the training group, training had no effect on brachial artery function (FMD) or structure (diameter and peak reactive hyperemia). These results suggest that LLBFRT may induce relevant vascular improvements in already well‐trained climbers, though its advantages over HLRT remain to be confirmed.

### 4.1 Brachial Artery Structure and Function

In the current study, climbers presented with high values of FMD% (~9%) and brachial artery baseline diameter (~4.4 mm), corresponding to the 80th‐90th percentile of reference values for healthy individuals of similar age [46, 47]. These findings support those from Thompson et al. [17] suggesting that climbers are already accustomed to the substantial shear stress fluctuations induced by repeated intermittent isometric contractions.

However, FMD remained unchanged after both training modalities supporting the “athlete paradox” concept [18, 48], wherein already trained individuals, including rock‐climbers [17], appear not to benefit from further training‐induced improvements in dilation capacity compared to untrained individuals. The elevated baseline FMD% (~9%) in our participants may thus have constrained potential gains. Additionally, this plateau in training effect may reflect the time‐course of arterial adaptations to exercise. While it is known that training increases FMD in ~2 to 5 weeks to adapt to the elevation of shear stress, the training stimulus also triggers structural vascular remodeling resulting in artery's resting diameter enlargement typically after ~4 to 6 weeks [14, 24] which, in turn, attenuates FMD% response [49]. Nevertheless, the allometrically scaled FMD (i.e., an analysis taking into account potential alterations in baseline diameter) was not altered either in the present study.

No brachial artery enlargement was detected following the training protocol. Our initial hypothesis of an increased diameter following the 5 weeks of intervention relied on the body of literature suggesting that LLBFRT can induce structural adaptations after ~4–8 weeks [23, 24, 25]. However, not only the vascular adaptations induced by training with venous restriction appear more important in older compared to younger active adults [31, 32], but increased conduit artery is also not universally observed [14, 33], and climbers already presented an enlarged resting brachial diameter before our LLBFRT protocol. These results suggest that the added vascular stimulus induced by LLBFRT was not important enough to lead to significant adaption of the conduit artery function or structure on already trained climbers, with little exposure to variations in shear stress and not having undergone structural vascular remodeling.

Furthermore, the amount of antegrade (i.e., positive) shear stress and its variations are positively correlated with change in FMD after both acute exercise and 2 weeks of training [14, 50, 51] whereas the amount of retrograde (i.e., negative) shear stress decreases FMD in a dose‐dependent manner [52]. If LLBFRT is often presented as a training modality causing high variations of shear stress due to the important hyperemic response during cuff deflation, it must be underlined that cuff inflation (at least at rest) reduces antegrade and increases retrograde shear rate [14, 52, 53, 54]. In our study, cuffs were inflated throughout the entire training session (~15 min) and only deflated at the end of the session, following recommendations to optimize muscular adaptations [19]. Therefore, it is conceivable that other BFR settings, with intermittent LLBFRT protocol may optimize vascular stimulus, by increasing total shear stress and its variations. However, the effect of an intermittent protocol will require further investigation as it may also increase retrograde shear stress.

In the current study, peak reactive hyperemia was unchanged following either LLBFRT or HLRT suggesting no adaptation in peripheral resistance vessels [44]. This finding is somewhat unexpected since enhanced capillarization [28], capillary filtration capacity [24, 26] and reactive hyperemia at both limb and microvascular levels [31, 55, 56] have been reported following LLBFRT. It is worth noting that methodologically, peak hyperemic response observed after arterial occlusion does not reach levels obtained following an ischemic exercise [43, 57]. Hence, our 5‐min occlusion protocol—aiming to assess FMD—may not have provided an optimal stimulus to detect the entire structural vascular adaptation in FD muscles. However, using plethysmography, these settings were sufficient to detect large improvements (~+30%) of the hyperemic response following calf LLBFRT in untrained healthy females [56]. Such discrepancy may also be attributed to the training status of our participants. Indeed, climbers demonstrated larger hyperemic response following a 5‐min handgrip ischemic exercise than non‐climbers [17] which likely make further improvements in resistance vessel structure and function more difficult to achieve in this population.

### 4.2 Vascular Responses to Exercise

#### 4.2.1 Blood Flow and Oxygenation

Brachial blood flow during FD contraction at low intensity (from 10% to 60% MVC) was improved by ~20% following LLBFRT and was associated with an increased blood volume (Hbtot) and O_2_ availability (HbO_2_) at the muscle level, consistent with the literature, albeit during lower body exercise [23, 35]. It should be noted that blood flow improvements were greater in LLBFRT compared to CON, but not compared to HLRT.

The apparent contradiction between the lack of changes in arterial structure and function, and the significant changes in blood flow following LLBFRT may simply reside in the nature of the stimuli (i.e., occlusion vs. exercise) and its amplitude (~4 times lower during our exercise). First, it is unlikely that these discrepancies may be explained by an improved pressor reflex, no study having reported an increase in blood‐pressure during exercise post LLBFRT protocol. Therefore, the increased blood flow during contraction is more likely to occur due to reduced peripheral resistances distal to the measurement site, that is, from better local vasodilatory response to exercise [58] or/and enhanced capillarization. The latter has been consistently reported following LLBFRT [26, 27, 28] and may be induced by an upregulation of vascular endothelial growth factor (VEGF) which is exacerbated following acute [59, 60] and chronic BFR training [27, 61, 62].

The primary stimuli known to increase muscle capillarity are shear stress, mechanical wall tension and metabolic stimuli like hypoxia [63]. The latter two are greatly exacerbated by LLBFRT, the former due to venous occlusion inducing upstream vessel hypertension [64], the second due to reduction in O_2_ delivery and limited metabolic clearance [22, 65]. These repeated stimuli most likely have accentuated the angiogenic and vasodilative response, which may have led to increased capillarization at the end of the training protocol, in turn allowing greater perfusion during contraction and increasing O_2_ availability to the exercising muscle. However, it remains speculative as capillarization has not been directly measured.

The enhanced brachial blood‐flow during exercise coupled with greater muscle O_2_ availability may contribute to increase muscle endurance following LLBFRT [35, 66]. Indeed, CF deeply relies on blood flow during isometric intermittent contractions, which cannot increase at intensities above CF. An improved blood supply may thus enhance total O_2_ delivery over the contraction–recovery cycle, potentially allowing for a greater CF. Similarly, greater preservation of muscle oxygenation suggests a delayed decline in oxidative metabolism that typically occurs when O_2_ availability becomes limited during sustained isometric efforts. Consequently, this may reduce the reliance on anaerobic ATP production pathways and delay muscle fatigue onset. However, such adaptations may only be effective at intensities below the MOT, that is, during low‐to‐moderate intensity phases of climbing routes.

#### 4.2.2 MOT

Our study was the first to determine FD MOT in climbers, which occurred at ~40% MVC and was characterized by large interindividual variability (from 15% to 70% MVC). Interestingly, this threshold appears lower than what has typically been reported in non‐climbers (~50%–60% MVC) [9, 10, 67, 68]. This discrepancy may stem from factors such as contraction specificity, participants' training status, and differences in the measurement method. Unlike traditional approaches using plethysmography or proximal artery Doppler, which assess global limb blood flow and may overestimate MOT, our method targets the oxygenation of the contracting muscle itself. However, our measure distal to the muscle may underestimate occlusion if the mechanical compression of the muscle is not homogenous.

Overall, MOT does not appear to be affected by LLBFRT in advanced to elite climbers, which may be surprising since it would be expected that a better vascular ability to overcome relatively high intramuscular pressure should also be measurable at lower intensities such as MOT. The marked interindividual variability, as well as the 5% steps used to determine MOT, may have contributed to blur the potential effects of training.

#### 4.2.3 Post‐Exercise Hyperemia

The hyperemic responses during recovery phases have great influence on CF or endurance during intermittent contractions [6, 7]. However, we did not directly assess brachial post‐contraction hyperemia due to the complexity of accurately measuring both during contraction and recovery phases, marked with important movement artifacts. Instead, we quantified muscle‐level blood flow using venous occlusion technique coupled with NIRS data. We demonstrated no effect of training on post contraction mBF. It may be noted here that our mBF determination was based on a single venous occlusion at each intensity, compared to 3 to 4 trials typically used for intermittent contractions [40, 69], having opted to perform measurements on a wider range of intensities. Nevertheless, future studies should focus more specifically on this post‐contraction hyperemic response following LLBFRT, both at conduit artery and muscular levels.

## 5 Limitations

There are some limitations to be acknowledged in our study. First, our brachial artery blood flow measurements during contractions were performed at a single time point (~30‐s). As a consequence, our results may not be fully transferable to other time points along the duration of the contraction, as some studies reported a gradual linear increase in blood flow in the conduit artery during isometric contractions at constant intensities from 20% to 100% MVC [68, 70]. However, LLBFRT has not been reported to affect the temporal kinetics of blood flow regulation [20, 71], and therefore measurement timing may only have a marginal effect, if any, on the observed training adaptations.

Second, large cuffs were used in this study so that LLBFRT compressed a large part of the biceps. It is well‐established that muscle adaptations are reduced or abolished in the cuff compression zone [35, 72]. It is possible that conduit artery adaptations were blunted in this area as ultrasonography measurements zone slightly overlapped with the distal edge of the compression zone, especially in small participants. However, we used an autoregulated BFR device which automatically adjusts cuff pressure during exercise to avoid pressure spikes due to muscle contraction. This technology has been shown to enhance LLBRT tolerability and safety [37, 73], but cannot be used with narrow cuffs. Hence, future studies are needed to differentiate vascular adaptations distal to the cuff vs. under the cuff.

Third, only males were included in this study. This choice was mainly due to the fact that women present larger variability in vascular responses due to ovarian cycle [74]. Women are highly underrepresented from LLBFRT research and several studies suggest that males and women present different muscular adaptation to LLBFRT [75, 76]. Hence, our results could not been directly generalized to female climbers and future studies should focus specifically on how women respond to LLBFRT [77].

Finally, participants were lying on their back, arms at heart level, in a pulling position with the arm positioned at 90° from the body axis in order to improve vascular measurement quality and reliability. This generalization of our blood flow results to a climbing‐specific position standing with arms up must therefore be done with caution. In addition, neither LLBFRT nor HLRT improved FD MVC. The low specificity of this position may explain the lack of muscle strength adaptations following training [78]. Indeed, FD strength is highly specific, as difference in FD strength between climbers and non‐climbers is lower or nonsignificant when measured on nonspecific tests [79]. Future studies evaluating muscle strength in a hanging position after FD LLBFRT are required.

## 6 Perspectives

Our study reports, for the first time, that 5 weeks of climbing‐specific finger flexor LLBFRT increased brachial blood flow and blood volume, and accentuated HbO_2_ reserves during low intensity contractions from 10% to 60% MVC in trained climbers. These adaptations occurred despite any measurable changes in endothelial function, conduit artery diameter, or post‐occlusive reactive hyperemia, suggesting small but relevant microvascular adaptations. Thus, even marginal improvements may contribute to finger flexors endurance improvements as previously demonstrated following LLBFRT and would be beneficial to athletes already at a high level of performance. Future studies should focus on: (i) quantifying hyperemic responses post‐contraction, which may be more pronounced and more generalizable to higher intensities; (ii) exploring vascular adaptations to intermittent LLBFRT which may optimize the vascular stimulus by increasing shear rate variations.

## Funding

This work is supported by the French National Research Agency in the framework of the “Investissements d'avenir” program (ANR‐15‐IDEX‐0002) and “PerfAnalytics” (ANR‐15‐IDEX‐0002). The funding organization had no influence on the collection of study data, their analysis and interpretation, or the writing of the manuscript.

## Ethics Statement

This study was approved by the French Ethics Committee for Research in Sports Science (IRB00012476‐2023‐10‐05‐246) and complied with the Declaration of Helsinki (2008).

## Consent

All participants provided written informed consent prior to their inclusion in the study and were fully informed about the experimental procedures, potential risks, and benefits.

## Conflicts of Interest

The authors declare no conflicts of interest.

## Supporting information


**Appendix S1:** sms70211‐sup‐0001‐AppendixS1.docx.

## Data Availability

The data that support the findings of this study are available from the corresponding author upon reasonable request.
