# Supplementary material for: Low‐Load Blood Flow Restriction Training Enhances Brachial Blood Flow During Exercise but not Reactive Hyperemia in Experienced Climbers
Source: Scand J Med Sci Sports. 2026 Jan 24;36(2):e70211. doi: 10.1111/sms.70211 (PMC12831503; doi:10.1111/sms.70211)
Supplement: Supplementary file 1 — Appendix S1: sms70211‐sup‐0001‐AppendixS1.docx. [file SMS-36-e70211-s001.docx]

**Supplementary Materials**

**Low-load blood flow restriction training enhances brachial blood flow during exercise but not reactive hyperemia in experienced climbers**

Titouan Paul Perrin^1^, Hugo Randy^1^, Pyrène Santal^1^, Xavier Hugues^2^, Nicolas Tourette^1^, Marie Coudurier^1^, Michel Guinot^1^, Violaine Cahouet^2^, Franck Quaine^2^, Stéphane Doutreleau^1^, Samuel Vergès^1^, Laurent Vigouroux^3^, Hugo Kerherve^4^, Mathieu Marillier*^1^ & Julien Vincent Brugniaux*^1^

*^1^Univ. Grenoble Alpes, Inserm, CHU Grenoble Alpes, HP2, 38000 Grenoble, France.*

*^2^Univ. Grenoble Alpes, CNRS, Grenoble INP, GIPSA-lab, 38000 Grenoble, France.*

*^3^Univ Aix-Marseille, CNRS, ISM, 13288 Marseille, France*

*^4^Univ Rennes, M2S - EA 7470, 35000 Rennes, France.*

* indicates senior co-authorship

**Methods**

Thirty-nine experienced male sport climbers were included in this study, of whom 36 completed the study, with three participants excluded due to injury during the training period. The flow chart of the study is presented in **Supplemental Figure 1**.


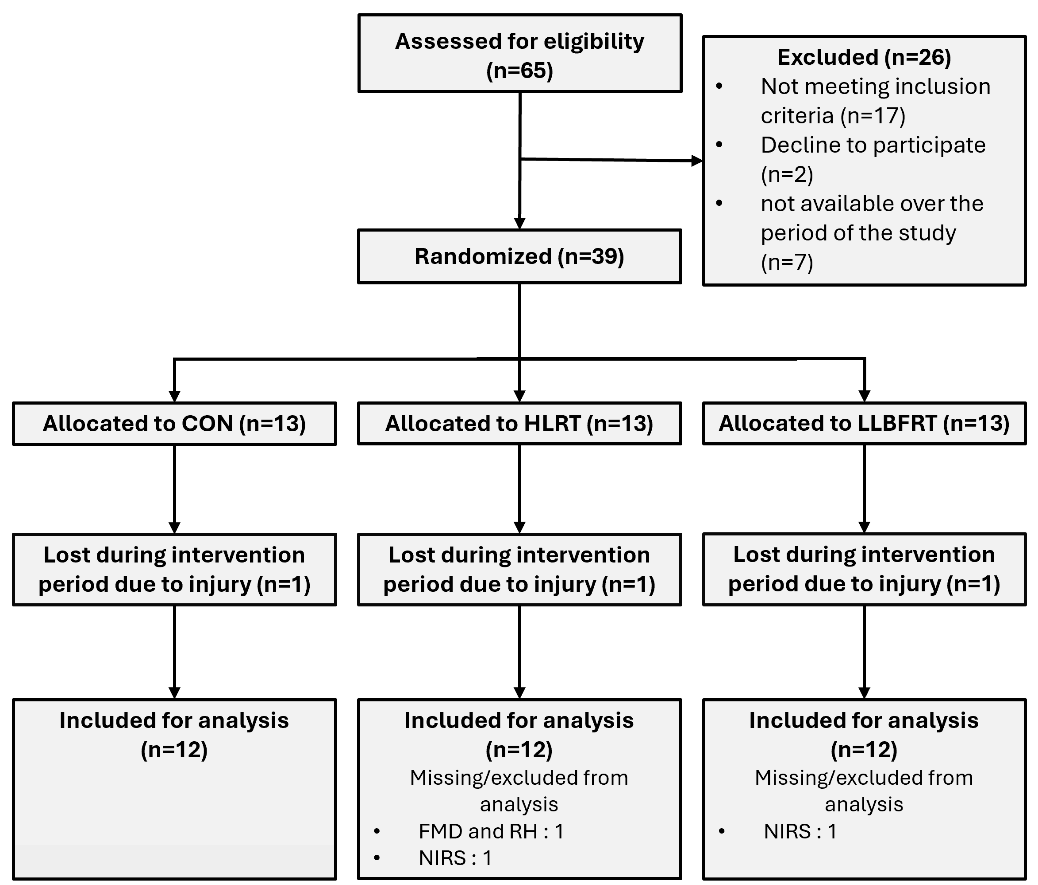


**Supplemental Figure 1.** **Study participants flow chart.**

Three participants got injured during the training protocol. One sustained a trapezoid injury during an experimental visit aiming to assess muscular adaptations (data presented elsewhere). The two others experienced pulley injuries during their climbing routines. Two participants were removed from NIRS analysis due to technical connection issues during the experimental visit. One participant was removed from FMD and RH analysis due to absence of blood flow occlusion (visually detected by an increase in HbO_2_ NIRS signal during the occlusion). CON: control group; FMD: flow-mediated dilation; HLRT: high-load resistance training group; LLBFRT: low-load blood flow restriction training group; NIRS: near-infrared spectroscopy; RH: reactive hyperemia.

**Results**

**Flow-mediated dilation (%) and peak reactive hyperemia**

There was no significant effect of training modality, of time or time × training group interaction for i) baseline diameter; ii) peak diameter; iii) Flow-mediated dilation (FMD%) (Supplemental Figure 2A); iv) FMD% corrected by shear rate or v) peak reactive hyperemia (Supplemental Figure 2B).


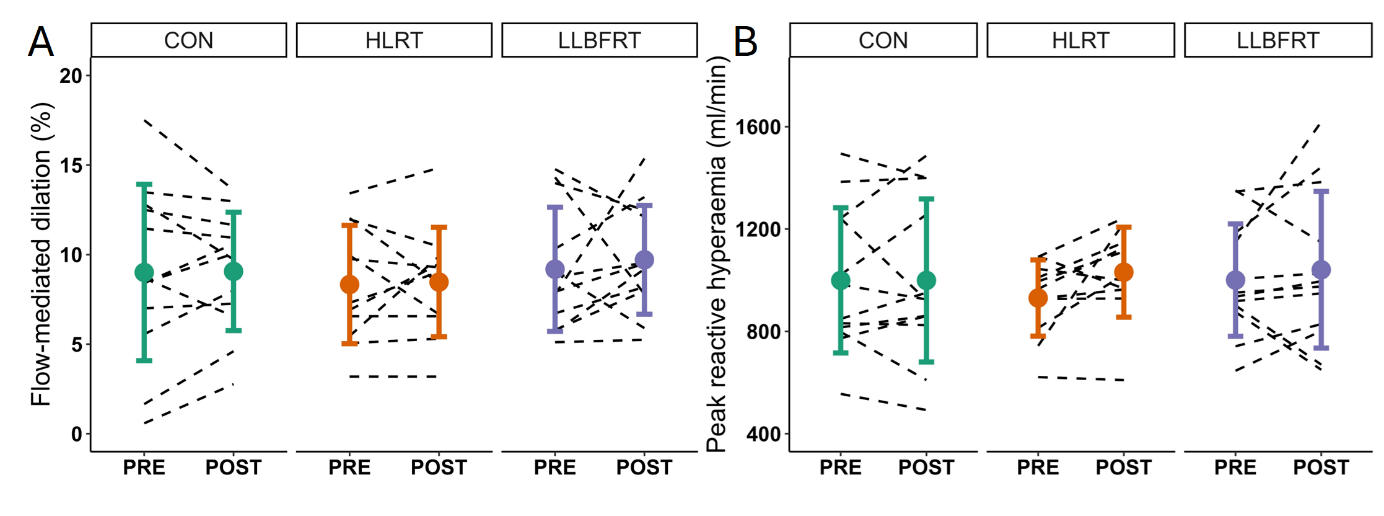
**Supplemental Figure 2.** **Effect of training on brachial artery flow-mediated dilation (A) and peak reactive hyperemia post cuff deflation (B).**

Data are represented as mean (filled circles) ± SD; individual responses are represented using dashed lines.

*Definition of abbreviations*. CON: control group; HLRT: high-load resistance training group; LLBFRT: low-load blood flow restricted training group; PRE: before training protocol, POST: After training protocol.

**Vascular and muscular responses to exercise**

Mean muscle occlusion threshold (MOT) and finger flexors (FD) maximal voluntary contraction (MVC) values are presented in Table 3 with individual values depicted in Supplemental Figure 3A and 3B. These variables did not change from PRE to POST, irrespective of the training modality.

Brachial artery responses during contractions from 10 to 60% MVC with ANOVA results are presented in Supplemental Table 1. In LLBFRT, there were significant time effects on brachial artery blood flow (*p=* 0.011) and blood velocity (*p=* 0.033) during contraction which were significantly increased by 19 ± 31% (*d*= 0.5) and 16 ± 30% (*d*= 0.41) in POST compared to PRE, respectively. However, there was no significant effect of time on artery diameter (p= 0.184). There was no time × contraction intensity interaction nor time effect on any variable in HLRT and CON.


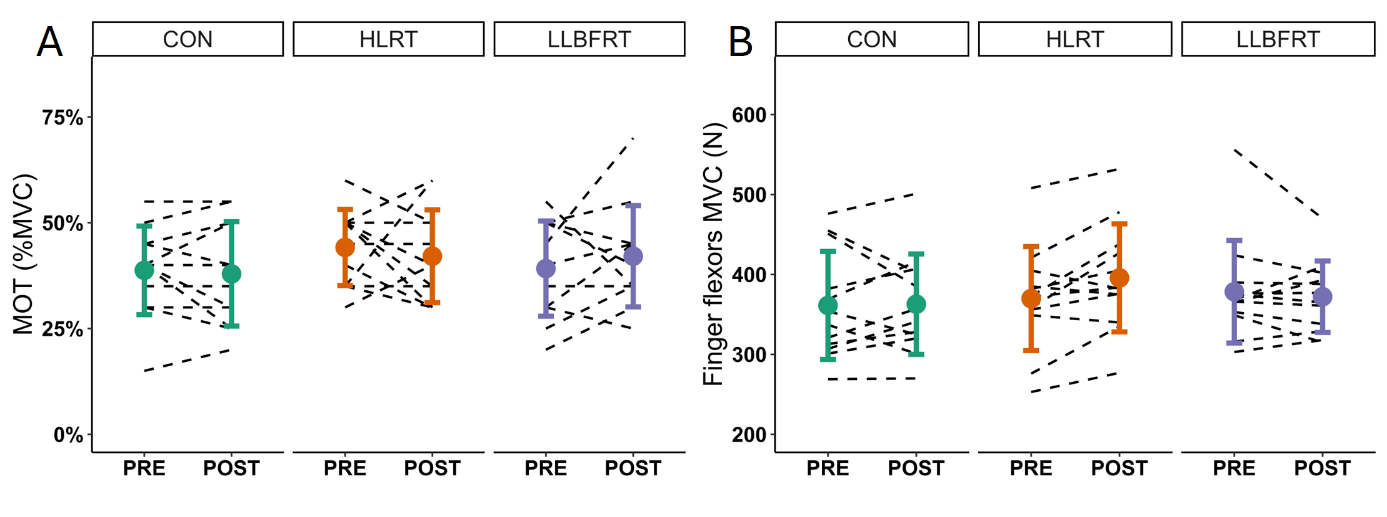
Considering differences between groups, ∆blood flow and ∆blood velocity from PRE to POST were significantly higher in LLBFRT than CON (*p=* 0.020 and *p=* 0.009) without difference between LLBFRT and HLRT (*p=* 0.331 and *p=* 0.34).

**Supplemental Figure 3. Effect of training on muscular occlusion threshold (A) and finger flexor MVC (B).**

Data are represented as mean (filled circles) ± SD; individual responses are represented using dashed lines.

*Definition of abbreviations*. CON: control group; HLRT: high-load resistance training group; LLBFRT: low-load blood flow restricted training group; MOT: muscular occlusion threshold; MVC: maximal voluntary contraction; PRE: before training protocol, POST: After training protocol.

**Supplemental Table 1. Brachial artery blood flow, diameter, and blood velocity during contractions across training modalities, contraction intensity and time points (PRE vs. POST).**

|  |  |  | Contraction intensity | | | | | | | ANOVA effects ($\eta_{p}^{2}$) | | |
| --- | --- | --- | --- | --- | --- | --- | --- | --- | --- | --- | --- | --- |
|  | Training modality | Time | 0%MVC | 10%MVC | 20%MVC | 30%MVC | 40%MVC | 50%MVC | 60%MVC | Time | Contraction intensity | Interaction |
| Brachial artery blood flow (ml/min) | CON | PRE | 212 ± 100 | 220 ± 54 | 218 ± 63 | 213 ± 63 | 188 ± 50 | 199 ± 65 | 178 ± 62 | 0.764 (0.009) | **0.023 (0.268)** | 0.770 (0.047) |
|  |  | POST | 220 ± 54 | 224 ± 52 | 223 ± 49 | 226 ± 56 | 183 ± 54 | 187 ± 69 | 198 ± 78 |  |  |  |
|  | HLRT | PRE | 200 ± 40 | 238 ± 60 | 245 ± 40 | 226 ± 57 | 204 ± 62 | 200 ± 56 | 187 ± 62 | 0.516 (0.039) | **0.008 (0.342)** | 0.629 (0.062) |
|  |  | POST | 239 ± 85 | 267 ± 76 | 257 ± 71 | 224 ± 75 | 232 ± 87 | 207 ± 70 | 214 ± 114 |  |  |  |
|  | LLBFRT | PRE | 248 ± 88 | 236 ± 69 | 227 ± 55 | 217 ± 79 | 208 ± 79 | 238 ± 97 | 217 ± 92 | **0.011 (0.460)** | 0.169 (0.151) | 0.438 (0.077) |
|  |  | POST | 276 ± 99 | 285 ± 97 | 259 ± 88 | 280 ± 100 | 241 ± 107 | 257 ± 107 | 244 ± 108 |  |  |  |
| Brachial artery diameter (cm) | CON | PRE | 0.43 ± 0.06 | 0.43 ± 0.04 | 0.43 ± 0.04 | 0.45 ± 0.05 | 0.44 ± 0.05 | 0.45 ± 0.04 | 0.45 ± 0.05 | 0.058 (0.289) | **<0.001 (0.432)** | 0.495 (0.076) |
|  |  | POST | 0.45 ± 0.04 | 0.44 ± 0.04 | 0.45 ± 0.04 | 0.45 ± 0.04 | 0.45 ± 0.04 | 0.45 ± 0.04 | 0.46 ± 0.04 |  |  |  |
|  | HLRT | PRE | 0.45 ± 0.02 | 0.45 ± 0.03 | 0.46 ± 0.04 | 0.46 ± 0.03 | 0.46 ± 0.03 | 0.47 ± 0.03 | 0.47 ± 0.03 | 0.359 (0.077) | **0.001 (0.362)** | 0.567 (0.068) |
|  |  | POST | 0.46 ± 0.03 | 0.46 ± 0.03 | 0.47 ± 0.04 | 0.47 ± 0.03 | 0.47 ± 0.04 | 0.47 ± 0.04 | 0.48 ± 0.03 |  |  |  |
|  | LLBFRT | PRE | 0.45 ± 0.05 | 0.44 ± 0.06 | 0.44 ± 0.05 | 0.44 ± 0.05 | 0.45 ± 0.06 | 0.46 ± 0.05 | 0.46 ± 0.05 | 0.184 (0.155) | **0.009 (0.278)** | 0.151 (0.148) |
|  |  | POST | 0.45 ± 0.05 | 0.45 ± 0.05 | 0.45 ± 0.06 | 0.46 ± 0.05 | 0.46 ± 0.05 | 0.45 ± 0.05 | 0.46 ± 0.06 |  |  |  |
| Brachial artery blood-velocity (cm/s) | CON | PRE | 22.8 ± 9.1 | 25 ± 6.9 | 25.2 ± 7.6 | 23 ± 7.0 | 20.4 ± 5.3 | 20.8 ± 7.0 | 18.1 ± 5.8 | 0.786 (0.007) | **0.003 (0.360)** | 0.860 (0.037) |
|  |  | POST | 22.9 ± 5.3 | 23.7 ± 4.2 | 23.4 ± 3.4 | 23 ± 4.0 | 19.1 ± 5.0 | 19.2 ± 7.1 | 19.3 ± 6.7 |  |  |  |
|  | HLRT | PRE | 20.4 ± 3.4 | 24 ± 3.6 | 24.5 ± 4.4 | 22.5 ± 5.2 | 20.4 ± 6.0 | 19.1 ± 5.2 | 18.3 ± 6.5 | 0.480 (0.046) | **0.001 (0.421)** | 0.477 (0.078) |
|  |  | POST | 23.7 ± 7.4 | 26.3 ± 6.2 | 24.6 ± 5.9 | 21.7 ± 6.9 | 22.3 ± 7.5 | 19.5 ± 5.9 | 19.3 ± 9.6 |  |  |  |
|  | LLBFRT | PRE | 26.4 ± 8.5 | 25.8 ± 7.3 | 25.2 ± 7.8 | 24.3 ± 10.8 | 22.5 ± 12.1 | 24.5 ± 11.0 | 22.9 ± 12.2 | **0.033 (0.352)** | 0.092 (0.200) | 0.682 (0.042) |
|  |  | POST | 28.1 ± 5.9 | 30.1 ± 8.1 | 27.8 ± 8.4 | 29.2 ± 11.3 | 24.3 ± 9.8 | 27.3 ± 11.2 | 25 ± 11.2 |  |  |  |

Data are mean ± SD. ANOVA effects are presented as p-value (partial-eta squared); Values are bolded when significant.

*Definition of abbreviations.* ANOVA: Analysis of variance; CON: control group; HLRT: high-load resistance exercising group; LLBFRT: low-load blood flow restricted group; MVC: maximum voluntary contraction.

**NIRS assessment during contractions**

NIRS responses during contractions from 10 to 60% MVC and post contraction mBF with ANOVA results are presented in Supplemental Table 2. In LLBFRT, there were significant time effects on HbO_2_ (*p=* 0.023) and Hbtot (*p=* 0.009). Regardless of contraction intensity, HbO2 (+ 4.3 ± 8.3 $\mu$mol, *d*=0.52) and Hbtot (+ 2.8 ± 5.4 $\mu$mol, *d*=0.52) were increased (or less decreased) during contraction in POST compared to PRE. In CON, there was significant effect of time on TSI (*p=* 0.014). Regardless of contraction intensity, TSI (+ 3.3 ± 6.9%, d=0.49) was less decreased during contraction in POST compared to PRE.

Regardless of training modality, mBF increased with contraction intensity (all *p*< 0.001) without any effect of Time (CON: *p=* 0.543; HLRT: *p=* 0.228; LLBFRT: *p=* 0.518). A time × contraction intensity interaction was detected on mBF only in CON (*p=* 0.046; HLRT: *p=* 0.410; LLBFRT: *p=* 0.359) but without any significant post-hoc.

**Supplemental Table 2. Variations of NIRS responses during contractions across training modalities, contraction intensity and time points (PRE vs. POST).**

| Variable | Training modality | Time | Contraction intensity | | | | | | ANOVA effect ($\eta_{p}^{2}$) | | |
| --- | --- | --- | --- | --- | --- | --- | --- | --- | --- | --- | --- |
|  |  |  | 10%MVC | 20%MVC | 30%MVC | 40%MVC | 50%MVC | 60%MVC | Time | Contraction  intensity | Interaction |
| HHb  (μmol) | CON | PRE | 2.8 ±  2.1 | 7.8 ±  7.1 | 14.2 ±  8.3 | 19.7 ± 11.5 | 23.2 ± 11.6 | 26.3 ± 11.4 | 0.095  (0.233) | **0.000**  **(0.817)** | 0.326  (0.097) |
|  |  | POST | 2.6 ±  1.5 | 5.8 ±  3.9 | 9.8 ±  5.2 | 15.3 ±  6.2 | 17.1 ±  7.7 | 20.7 ±  7.9 |  |  |  |
|  | HLRT | PRE | 3.0 ±  3.4 | 7.0 ±  7.5 | 14.2 ± 12.6 | 20.6 ± 11.4 | 21.9 ± 11.5 | 23.7 ± 11.2 | 0.421  (0.066) | **0.000**  **(0.754)** | 0.093  (0.167) |
|  |  | POST | 3.2 ±  2.9 | 7.5 ±  7.5 | 15.5 ± 10.8 | 14.8 ± 10.4 | 21.0 ± 12.7 | 21.5 ± 11.2 |  |  |  |
|  | LLBFRT | PRE | 3.6 ±  2.7 | 10.9 ±  8.0 | 18.2 ± 10.9 | 25.6 ± 10.1 | 27.4 ± 10.1 | 29.8 ±  9.0 | 0.383  (0.077) | **0.000**  **(0.796)** | 0.480  (0.084) |
|  |  | POST | 4.6 ±  3.7 | 10.6 ±  7.3 | 16.5 ± 10.5 | 21.1 ± 13.0 | 25.5 ± 12.0 | 28.2 ± 10.3 |  |  |  |
| HbO_2_  (μmol) | CON | PRE | -3.0 ±  5.6 | -6.8 ±  5.3 | -12.9 ±  9.0 | -22.0 ± 11.2 | -28.5 ± 11.3 | -33.6 ± 11.8 | 0.096  (0.232) | **0.000**  **(0.852)** | 0.181  (0.140) |
|  |  | POST | -2.4±  2.5 | -4.3 ±  4.9 | -5.7 ±  8.4 | -14.0 ± 7.8 | -21.6 ± 11.0 | -32.1 ± 12.1 |  |  |  |
|  | HLRT | PRE | -2.5 ±  1.9 | -4.7 ±  5.2 | -10.1 ±  9.6 | -18.6 ± 13.7 | -26.0 ± 15.8 | -36.0 ± 12.2 | 0.135  (0.209) | **0.000**  **(0.792)** | **0.001**  **(0.327)** |
|  |  | POST | -1.5 ±  2.0 | -4.4 ±  5.2 | -12.1 ± 9.8 | -15.1 ± 13.9 | -23.3 ± 14.5 | -27.6 ± 12.1 |  |  |  |
|  | LLBFRT | PRE | -3.0 ±  2.4 | -9.7 ±  7.7 | -17.6 ± 12.6 | -26.9 ± 12.7 | -31.8 ± 12.9 | -35.8 ± 11.6 | **0.023**  **(-0.416)** | **0.000**  **(0.835)** | 0.134  (0.180) |
|  |  | POST | -2.7 ±  2.9 | -6.3 ±  4.2 | -13.2 ± 9.2 | -18.5 ± 13.0 | -24.8 ± 14.2 | -33.5 ± 12.0 |  |  |  |
| Hbtot  (μmol) | CON | PRE | -0.2 ±  5.9 | 1.0 ±  5.0 | 1.3 ±  5.6 | -2.3 ±  6.0 | -5.4 ±  3.1 | -7.2 ±  4.2 | 0.588  (0.028) | **0.000**  **(0.693)** | **0.009**  **(0.238)** |
|  |  | POST | 0.2 ±  2.9 | 1.6 ±  3.8 | 4.1 ±  4.3 | 1.3 ±  4.5 | -4.6 ±  6.0 | -11.3 ± 6.1 |  |  |  |
|  | HLRT | PRE | 0.5 ±  2.3 | 2.3 ±  4.1 | 4.2 ±  5.8 | 2.0 ±  8.7 | -4.1 ±  9.4 | -12.3 ±  7.3 | 0.324  (0.097) | **0.000**  **(0.576)** | **0.007**  **(0.267)** |
|  |  | POST | 1.7 ±  1.9 | 3.1 ±  3.9 | 3.4 ±  6.4 | -0.3 ±  6.9 | -2.2 ±  6.9 | -6.2 ±  5.6 |  |  |  |
|  | LLBFRT | PRE | 0.6 ±  2.2 | 1.2 ±  2.3 | 0.6 ±  3.3 | -1.3 ±  6.6 | -4.4 ±  6.6 | -6.0 ±  5.2 | **0.009**  **(0.509)** | **0.002**  **(0.484)** | 0.340  (0.104) |
|  |  | POST | 1.9 ±  2.1 | 4.4 ±  4.6 | 3.3 ±  2.8 | 2.6 ±  3.7 | 0.7 ±  6.1 | -5.3 ±  7.2 |  |  |  |
| TSI  (%) | CON | PRE | -2.5 ±  1.8 | -6.9 ±  5.9 | -13.9 ± 7.4 | -21.6 ± 9.9 | -27.0 ±  8.3 | -31.4 ±  8.8 | **0.014**  **(0.376)** | **0.000**  **(0.869)** | 0.086  (0.196) |
|  |  | POST | -2.4 ±  1.8 | -6.1 ±  3.7 | -10.2 ± 7.5 | -17.7 ± 8.7 | -19.7 ±  8.0 | -27.1 ± 7.4 |  |  |  |
|  | HLRT | PRE | -2.7 ±  2.8 | -7.5 ±  6.9 | -12.6 ± 9.4 | -20.2 ± 10.6 | -23.8 ± 11.4 | -29.3 ± 9.7 | 0.583  (0.031) | **0.000**  **(0.811)** | 0.119  (0.157) |
|  |  | POST | -2.9 ±  2.2 | -7.5 ±  7.5 | -15.7 ± 11.7 | -16.2 ± 12.4 | -24.2 ± 16.2 | -25.3 ± 9.7 |  |  |  |
|  | LLBFRT | PRE | -4.1 ±  2.4 | -11.6 ± 9.2 | -19.2 ± 11.5 | -27.5 ± 14.8 | -29.0 ± 13.9 | -31.0 ± 13.4 | 0.443  (0.060) | **0.000**  **(0.831)** | 0.423  (0.082) |
|  |  | POST | -5.0 ±  4.1 | -9.5 ±  5.9 | -16.4 ±  8.7 | -21.9 ± 10.7 | -27.2 ± 10.9 | -31.1 ± 9.1 |  |  |  |
| mBF  (ml.min^-1^) | CON | PRE | 15.9 ±  7.5 | 25.8 ±  9.2 | 27.1 ± 11.6 | 40.9 ± 19.2 | 39.30 ± 15.6 | 43.9 ± 14.3 | 0.543 (0.035) | **<0.001 (0.734)** | **0.046 (0.208)** |
|  |  | POST | 23.3 ± 18.5 | 24.8 ± 17.3 | 30.6 ± 19.8 | 36.8 ± 25.0 | 35.1 ± 21.3 | 39.1 ± 27.2 |  |  |  |
|  | HLRT | PRE | 17.4 ± 11.8 | 22.1 ± 14.0 | 28.4 ± 15.0 | 34.2 ± 21.8 | 35.7 ± 28.3 | 41.6 ± 31.1 | 0.228 (0.141) | **<0.001 (0.716)** | 0.410 (0.090) |
|  |  | POST | 24.3 ± 17.4 | 31.2 ± 20.9 | 36.4 ± 20.5 | 36.2 ± 26.5 | 47.6 ± 25.2 | 46.7 ± 26.6 |  |  |  |
|  | LLBFRT | PRE | 14.7 ±  8.4 | 19.2 ± 10.3 | 22.2 ±  8.2 | 30.5 ± 14.1 | 30.7 ± 14.2 | 29.8 ±  8.7 | 0.518 (0.043) | **<0.001 (0.643)** | 0.359 (0.099) |
|  |  | POST | 18.5 ± 10.4 | 25.8 ± 12.6 | 29.6 ± 19.5 | 30.1 ± 20.9 | 34.9 ± 20.1 | 33.1 ± 17.5 |  |  |  |

Data are mean ± SD. Each value corresponds to the variation of the variable from the 5^th^ to the 25^th^ second of contraction, except for mBF with is calculated from the Hbtot increase during the first second of venous occlusion, ten seconds after each contraction. ANOVA effects are presented as p-value (partial-eta squared); Values are bolded when significant.

*Definition of abbreviations.* ANOVA: Analysis of variance; CON: control group; HbO_2_: oxy-hemoglobin; Hbtot: Total hemoglobin; HHb: deoxy-hemoglobin; HLRT: high-load resistance exercising group; LLBFRT: low-load blood flow restricted group; mBF: muscle blood flow estimated by near-infrared spectroscopy with venous occlusion technique; MVC: maximal voluntary contraction; TSI: Tissue saturation index.
